# Association of the Functional Medicine Model of Care With Patient-Reported Health-Related Quality-of-Life Outcomes

**DOI:** 10.1001/jamanetworkopen.2019.14017

**Published:** 2019-10-25

**Authors:** Michelle Beidelschies, Marilyn Alejandro-Rodriguez, Xinge Ji, Brittany Lapin, Patrick Hanaway, Michael B. Rothberg

**Affiliations:** 1 Center for Functional Medicine, Cleveland Clinic, Cleveland, Ohio; 2 Quantitative Health Sciences, Cleveland Clinic, Cleveland, Ohio; 3 Institute for Functional Medicine, Federal Way, Washington; 4 Community Care, Cleveland Clinic, Cleveland, Ohio

## Abstract

**Importance:**

The incidence of chronic disease is increasing along with health care–related costs. The functional medicine model of care provides a unique operating system to reverse illness, promote health, and optimize function. The association between this model of care and patient’s health-related quality of life (HRQoL) is unknown.

**Objective:**

To assess the association between functional medicine and patient-reported HRQoL using Patient-Reported Outcome Measurement Information System (PROMIS) global health measures.

**Design, Setting, and Participants:**

A retrospective cohort study was performed to compare 7252 patients aged 18 years or older treated in a functional medicine setting with propensity score (PS)–matched patients in a primary care setting. Sensitivity analyses assessed improvement limited to patients seen at both 6 and 12 months. The study included patients who visited the Cleveland Clinic Center for Functional Medicine or a Cleveland Clinic family health center between April 1, 2015, and March 1, 2017.

**Main Outcomes and Measures:**

The primary outcome was change in PROMIS Global Physical Health (GPH) at 6 months. Secondary outcomes included PROMIS Global Mental Health (GMH) at 6 months and PROMIS GPH and GMH at 12 months. The PROMIS GPH and GMH scores were transformed to a *T*-score from 0 to 100 with a mean of 50. Higher scores indicate a better health-related quality of life.

**Results:**

Of the 7252 patients (functional medicine center: 1595; family health center: 5657), 4780 (65.9%) were women; mean (SD) age was 54.1 (16.0) years. At 6 months, functional medicine patients exhibited significantly larger improvements in PROMIS GPH *T*-score points than were seen in patients treated at a family health center (mean [SD] change, functional medicine center: 1.59 [6.29] vs family health center: 0.33 [6.09], *P* = .004 in 398 PS-matched pairs). At 12 months, functional medicine patients showed improvement similar to that observed at 6 months; however, comparisons with patients seen at the family health center were not significant. Patients in the functional medicine center with data at both 6 and 12 months demonstrated improvements in PROMIS GPH (mean [SD], 2.61 [6.53]) that were significantly larger compared with patients seen at a family health center (mean [SD], 0.25 [6.54]) (*P* = .02 in 91 PS-matched pairs).

**Conclusions and Relevance:**

In this study, the functional medicine model of care demonstrated beneficial and sustainable associations with patient-reported HRQoL. Prospective studies are warranted to confirm these findings.

## Key Points

**Question:**

Is the functional medicine model of care associated with patient-reported health-related quality of life?

**Findings:**

In this cohort study of 7252 eligible patients (functional medicine center: 1595; family health center: 5657), functional medicine patients exhibited significantly larger improvements in Patient-Reported Outcome Measurement Information System Global Physical Health at 6 months than propensity-matched patients at a family health center (398 matched pairs). Improvements in Patient-Reported Outcome Measurement Information System Global Physical Health appeared to be sustained at 12 months but not significantly different from those at the family health center.

**Meaning:**

The findings of this study suggest that functional medicine may have the ability to improve global health in patients.

## Teaser

This cohort study uses the Patient-Reported Outcome Measurement Information System to assess the association between the functional medicine model of care and patient-reported health-related quality of life among adults.

## Introduction

Chronic disease is challenging health in the United States with nearly 100 million people having 1 or more chronic conditions in 2014.^1^ These individuals contribute to 90% of the nation’s annual health care expenditure.^1^ Chronic disease is a major contributor to health care costs owing to the need for disease management^2^ and care for elderly individuals.^3^ Without new approaches that focus on reversing chronic disease, our current health care model will become economically unsustainable.^4^

Nutrition and lifestyle choices can be used to manage chronic disease^5^; however, their use as a first-line therapy has historically been challenging for primary care physicians because most feel underequipped to deliver lifestyle recommendations^6^ despite the fact that nutrition and lifestyle are a foundation for most guidelines. There are various reasons for this feeling of inadequate preparation, including nutrition education level,^7^ confidence in the available nutrition evidence,^8,9^ and time with the patient.

Moreover, many chronic diseases are not diseases per se, but rather descriptions of symptoms or laboratory abnormalities. Conventional care is focused on managing symptoms of disease (eg, hypertension, abnormal blood glucose level), but underlying causes are rarely identified.

The functional medicine model of care provides an operating system that works to reverse illness, promote health, and optimize function by addressing underlying causes, symptoms, and functional imbalances in interconnected biological networks.^10^ These imbalances may impair principal biological functions (assimilation, defense and repair, energy production, biotransformation, communication, transport, and structural integrity) that result from gene-environment interactions, including lifestyle, environmental toxins, and the microbiome. Functional medicine removes triggers for illness and provides inputs to restore and optimize health. Functional medicine also addresses social determinants, including the psychological, emotional, and spiritual aspects of health and disease.^11^ A foundation of functional medicine is the use of food as medicine to prevent, treat, and reverse chronic disease. The functional medicine model of care may have the ability to improve patient’s health-related quality of life (HRQoL), including physical function and well-being. Therefore, the purpose of the present study was to investigate the association between the functional medicine model of care and HRQoL by comparing functional medicine with care received in a family medicine setting.

## Methods

### Study Design and Population

**Figure 1.**
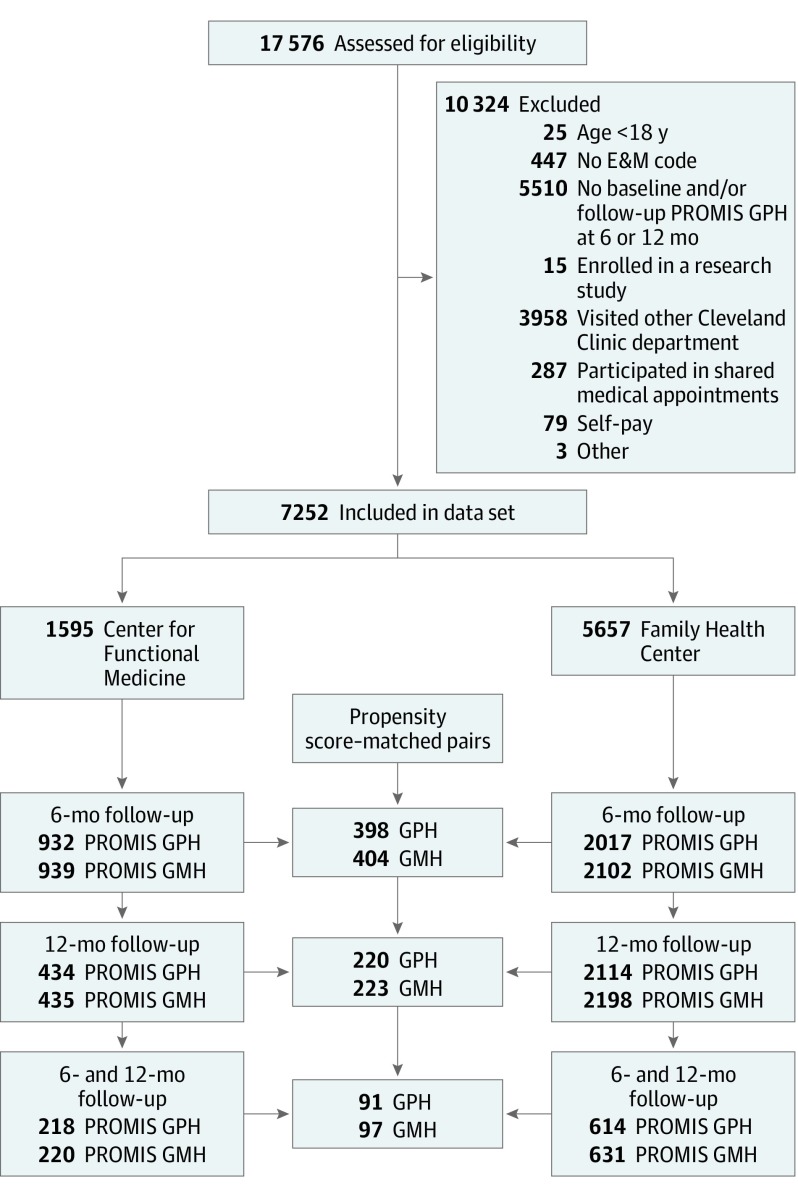
Study Flow Diagram E&M indicates evaluation and management; GMH, Global Mental Health; GPH, Global Physical Health; and PROMIS, Patient-Reported Outcome Measurement Information System.

A single-center retrospective cohort study was conducted to evaluate the longitudinal association of HRQoL in patients seen at Cleveland Clinic Center for Functional Medicine (hereafter, Center for Functional Medicine) vs receiving primary care at Cleveland Clinic Twinsburg Family Health Center (hereafter, Family Health Center). Figure 1 summarizes the study design and exclusion criteria. Patients were eligible for the study if they were 18 years or older and visited a clinician at the Center for Functional Medicine or the Family Health Center between April 1, 2015, and March 1, 2017. Patients must also have had a baseline Patient-Reported Outcome Measurement Information System (PROMIS) Global Physical Health (GPH) score and at least 1 follow-up score determined within a year of their initial visit, either at 6 months (mean [SD], 182 [30] days) or at 12 months (365 [30] days). This study followed the Strengthening the Reporting of Observational Studies in Epidemiology (STROBE) reporting guideline for cohort studies.^12^ The study protocol was reviewed and approved by the institutional review board of Cleveland Clinic Foundation in Cleveland, Ohio. Because this was a minimal risk study using data collected for routine clinical practice, a waiver of informed consent and Health Insurance Portability and Accountability Act authorization was granted.

### Data Source and Measures

The Knowledge Program Data Registry of Cleveland Clinic provided the data used in these analyses.^13^ The Knowledge Program Data Registry was responsible for the systematic collection of patient-reported outcomes at all Cleveland Clinic sites throughout the study period.^13^ Data on patient demographics and comorbidities were obtained from the electronic health record and race/ethnicity was typically self-reported. Data were deidentified and then securely stored. Approximate household income was estimated using the median income by zip code based on 2010 census data.

Patients’ HRQoL was measured using PROMIS Scale, version 1.2 Global Health (PROMIS GH). PROMIS, funded by the National Institutes of Health Roadmap Initiative, is a psychometrically validated dynamic system that measures self-reported health across multiple domains in patients with a wide range of diseases and demographic characteristics.^14^ PROMIS GH is a set of self-administered questions that measure physical, mental, and social health, and it provides a measure of overall health.^15,16^ Higher scores indicate a better health-related quality of life. PROMIS GH comprises 10 items and produces 2 summary scores: Global Physical Health (GPH) and Global Mental Health (GMH). The GPH measure includes 4 items on physical health, physical functioning, pain intensity, and fatigue, whereas, the GMH comprises 4 items on overall quality of life, mental health, satisfaction with social activities, and emotional problems. PROMIS physical function measures are sensitive enough to detect longitudinal changes due to targeted clinical interventions and able to distinguish among diverse chronic diseases.^17,18^ Summary scores are centered on the 2000 US Census with respect to age, sex, educational level, and race/ethnicity and are transformed to a *T*-score with a mean (SD) of 50 (10).^14^ Changes of 5 points suggest a meaningful or clinically important change; higher scores indicate a better HRQoL.^19,20^ Patients were prompted to complete scores at each visit either through the patient portal before the visit or in the waiting room using a tablet. Patients visiting the Center for Functional Medicine were typically encouraged to schedule follow-up visits every 3 months, as needed, for up to 1 year. Patients without scores at specific time points were excluded from the present study. PROMIS GPH and GMH scores were examined at baseline (initial visit), 6 months (follow-up visit), and 12 months (follow-up visit) based on clinical relevance.

Our primary outcome was change in PROMIS GPH scores from baseline to 6 months. Secondary outcomes included change in GPH scores from baseline to 12 months, as well as change in GMH scores at 6 months and 12 months.

### Statistical Analysis

Descriptive statistics are reported for all patients in the study cohort. Demographics, diagnostic category, and baseline PROMIS GH scores were summarized using frequency count with percentage for categorical variables and mean (SD) or median with interquartile range for continuous variables, as appropriate. Characteristics were compared across groups using the χ^2^ test for categorical variables and a 2-tailed, unpaired *t* test or Mann-Whitney test, as appropriate, for continuous variables. Characteristics were also compared for patients included in and excluded from the study (eTable 1 in the Supplement). Diagnostic categories were organized based on *International Classification of Diseases, Ninth Revision*, and *International Statistical Classification of Diseases and Related Health Problems, Tenth Revision*, diagnoses (eTable 2 in the Supplement).

Because patients seen in functional medicine differ from those in primary care, propensity score (PS) matching was used to balance the baseline differences in demographics and other characteristics between the 2 groups. Propensity scores for the probability of being seen in the Center for Functional Medicine vs the Family Health Center were estimated with multivariable logistic regression, including variables that differ by location: age, sex, race/ethnicity, marital status, income, baseline PROMIS score, comorbidities (ie, diabetes, depression, and hypertension), total number of visits within the past 12 months, and diagnostic category. Missing data were imputed under fully conditional specification using the default settings of the Multiple Imputation by Chained Equations, version 2.13 package.^21^ Propensity score matching was implemented using the R package Matching (R Foundation). A 1:1 match was performed with nonreplacement and a caliper of 0.2. Baseline characteristics and outcomes were compared between groups before and after PS matching using standardized differences, with differences less than 10% considered acceptable.^22^ Because all measured characteristics were balanced in the PS-matched cohorts, no further adjustments were made in determining the difference in PROMIS GPH or GMH scores.

Outcomes of patients seen in the Center for Functional Medicine and those seen in the Family Health Center were compared using a paired *t* test. The proportion of patients who improved GPH or GMH scores by 5 or more points, defining clinically meaningful change, was examined using the McNemar test. Based on the difference in proportions of patients reaching meaningful improvement, the number needed to treat was calculated.

Sensitivity analyses were conducted to explore the association of nonresponse bias. The GPH and GMH measures were limited to patients who had scores available at both 6 and 12 months. Analyses were conducted as described above within these groups.

Statistical analyses were conducted using SAS, version 9.4 (SAS Institute Inc) and R, version 3.2.4. Statistical significance was established at *P* < .05.

## Results

In total, 7252 new patients (Family Health Center: 5657 and Center for Functional Medicine: 1595) were included in the present study (Figure 1). Mean (SD) age of all patients was 54.1 (16.0) years, 4780 (65.9%) were women, and 6383 individuals (86.6%) were white. Table 1 reports the cohort characteristics prior to PS matching. Compared with patients seen at the Center for Functional Medicine, patients seen at the Family Health Center had a higher median (interquartile range [IQR]) income ($72 874.0 [IQR, $55 657.0-$82 802.0]; vs $59 286.0 [IQR, $45 787.0-$72 874.0]; *P* < .001), higher mean baseline PROMIS GPH scores (mean [SD], 48.75 [8.38] vs 44.81 [8.10]; *P* < .001) and PROMIS GMH scores (mean [SD], 50.27 [9.08] vs 44.89 [8.88]; *P* < .001), and higher prevalence of diabetes (1930 of 5657 [34.1%] vs 285 of 1595 [17.9%]; *P* < .001) and hypertension (2881 of 5657 [50.9%] vs 306 of 1595 [19.2%]; *P* < .001). Missing data were minimal (<2%) and imputed before PS matching. After PS matching, there were 398 patients in each group and there were no differences in any characteristic included in the PS (Table 2). A comparison of patients included in the analyses vs those excluded appears in eTable 1 in the Supplement.

**Table 1.** Cohort Characteristics of 7252 Patients by Center

Characteristic	No. (%)		*P* Value
	Center for Functional Medicine	Family Health Center	
Patients, No.	1595	5657	
Age, mean (SD), y	49.4 (14.1)	55.4 (16.2)	<.001
Women	1300 (81.5)	3480 (61.5)	<.001
White race	1474 (92.4)	4809 (85.0)	<.001
Married	1101 (69.0)	3652 (64.6)	.002
Household income, median (IQR), $	59 286.0 (45 787.0-72 874.0)	72 874.0 (55 657.0-82 802.0)	<.001
Diabetes	285 (17.9)	1930 (34.1)	<.001
Depression	411 (25.8)	1362 (24.1)	.18
Hypertension	306 (19.2)	2881 (50.9)	<.001
Baseline score, mean (SD)			
PROMIS GPH	44.81 (8.10)	48.75 (8.38)	<.001
PROMIS GMH	44.89 (8.88)	50.27 (9.08)	<.001
Total visits, mean (SD) No.	5.31 (3.01)	4.13 (2.29)	<.001
Functional medicine diagnostic category^a^			
Infection	250 (15.7)	278 (4.9)	<.001
Autoimmune	391 (24.5)	334 (5.9)	<.001
Allergen	119 (7.5)	68 (1.2)	<.001
Cancer	111 (7.0)	247 (4.4)	<.001
Hormones	790 (49.5)	1255 (22.2)	<.001
Energy mitochondria	756 (47.4)	96 (1.7)	<.001
Nutrition	43 (2.7)	53 (0.9)	<.001
Mood	79 (5.0)	94 (1.7)	<.001
Neurology	437 (27.4)	305 (5.4)	<.001
HEENT	50 (3.1)	137 (2.4)	.13
CVD	183 (11.5)	1165 (20.6)	<.001
Gut	657 (41.2)	358 (6.3)	<.001
Skin	273 (17.1)	212 (3.7)	<.001
Structure	374 (23.4)	620 (11.0)	<.001
Genitourinary	352 (22.1)	357 (6.3)	<.001
Trauma	10 (0.6)	66 (1.2)	.08

Abbreviations: Center for Functional Medicine, Cleveland Clinic Center for Functional Medicine; CVD, cardiovascular disease; Family Health Center, Cleveland Clinic Twinsburg Family Health Center; HEENT, head, eyes, ears, nose, and throat; IQR, interquartile range; PROMIS GMH, Patient-Reported Outcome Measurement Information System Global Mental Health; PROMIS GPH, PROMIS Global Physical Health.

^a^ Definitions provided in eTable 2 in the Supplement.

**Table 2.** Characteristics of Propensity Score–Matched Patients With PROMIS GPH Scores at 6 Months

Characteristic	No. (%)		Standardized Difference^a^
	Center for Functional Medicine	Family Health Center	
Patients, No.	398	398	
Age, mean (SD)	52.70 (13.54)	51.81 (16.25)	0.06
Women	302 (75.9)	301 (75.6)	0.006
White race	363 (91.2)	363 (91.2)	0.001
Married	267 (67.1)	272 (68.3)	0.027
Household income, median (IQR), $	62 776.0 (48 244.0-76 831.0)	65 052.0 (46 432.0-72 874.0)	0.056
Diabetes	100 (25.1)	97 (24.4)	0.017
Depression	118 (29.6)	104 (26.1)	0.078
Hypertension	107 (26.9)	104 (26.1)	0.017
Baseline score, mean (SD)			
PROMIS GPH	46.18 (8.67)	46.30 (8.85)	0.014
PROMIS GMH^b^	46.37 (8.98)	47.88 (9.29)	0.165
Total visits, mean (SD), No.	2.58 (0.94)	2.59 (1.28)	0.004
Functional medicine diagnostic category^c^			
Infection	27 (6.8)	26 (6.5)	0.01
Autoimmune	55 (13.8)	52 (13.1)	0.022
Allergen	14 (3.5)	12 (3.0)	0.028
Cancer	22 (5.5)	16 (4.0)	0.071
Hormones	116 (29.1)	108 (27.1)	0.045
Energy mitochondria	41 (10.3)	34 (8.5)	0.06
Nutrition	4 (1.0)	5 (1.3)	0.024
Mood	6 (1.5)	6 (1.5)	0.001
Neurology	59 (14.8)	53 (13.3)	0.043
HEENT	8 (2.0)	11 (2.8)	0.049
CVD	51 (12.8)	44 (11.1)	0.054
Gut	64 (16.1)	58 (14.6)	0.042
Skin	22 (5.5)	23 (5.8)	0.011
Structure	64 (16.1)	57 (14.3)	0.049
Genitourinary	42 (10.6)	38 (9.5)	0.033
Trauma	2 (0.5)	3 (0.8)	0.032

Abbreviations: Center for Functional Medicine, Cleveland Clinic Center for Functional Medicine; CVD, cardiovascular disease; Family Health Center, Cleveland Clinic Twinsburg Family Health Center; HEENT, head, eyes, ears, nose, and throat; IQR, interquartile range; PROMIS GMH, Patient-Reported Outcome Measurement Information System Global Mental Health; PROMIS GPH, PROMIS Global Physical Health.

^a^ Difference in means or proportions divided by SE; imbalance defined as absolute value greater than 0.10.

^b^ Not included in propensity score match.

^c^ Definitions provided in eTable 2 in the Supplement.

Table 3 reports the changes in PROMIS GPH and GMH scores in PS-matched cohorts. At 6 months, patients seen at the Center for Functional Medicine had a significant improvement in their PROMIS GPH scores from 46.18 (8.67) at baseline to 47.77 (8.15) at 6 months in 398 patients (*P* < .001). The PROMIS GPH mean (SD) change at 6 months was also significantly greater than that seen in patients treated at the Family Health Center (Center for Functional Medicine: 1.59 [6.29] vs Family Health Center: 0.33 [6.09] *T*-score points in 398 patients; *P* = .004). In addition, more patients seen at the Center for Functional Medicine improved their PROMIS GPH scores by 5 or more points than those seen at the Family Health Center (Center for Functional Medicine: 123 [30.9%] vs Family Health Center: 88 [22.1%]; *P* = .006; number needed to treat, 11). At 12 months, patients at the Center for Functional Medicine showed improvement in PROMIS GPH similar to that observed at 6 months (from 45.90 [8.33] at baseline to 47.50 [8.49] at 12 months in 220 patients; *P* < .001); however, comparisons with the Family Health Center were not significant. Categorical improvements of PROMIS GPH scores from baseline to 6 months are displayed in the eFigure in the Supplement.

**Table 3.** Changes in PROMIS GPH and GMH *T*-Scores Over Time by Propensity Score–Matched Group

Outcome	Mean (SD)		Difference in Difference (SE)	*P* Value for Comparison
	Center for Functional Medicine	Family Health Center		
**PROMIS GPH**				
Baseline to 6 mo, No.^a^	398	398	NA	NA
*T*-score				
Baseline	46.18 (8.67)	46.30 (8.85)	NA	.85
6 mo	47.77 (8.15)	46.63 (8.69)	NA	.049
Change	1.59 (6.29)^b^	0.33 (6.09)	+1.26 (0.58)	.004
Improve ≥5 points, No. (%)	123 (30.9)	88 (22.1)	NA	.006
Worsen ≥5 points, No. (%)	59 (14.8)	69 (17.3)	NA	.40
Baseline to 12 mo, No.	220	220	NA	NA
*T*-score				
Baseline	45.90 (8.33)	44.67 (8.48)	NA	.12
12 mo	47.50 (8.49)	45.76 (9.09)	NA	.04
Change	1.60 (6.05)^b^	1.09 (6.57)	+0.51 (0.81)	.41
Improve ≥5 points, No. (%)	54 (24.5)	56 (25.5)	NA	.91
Worsen ≥5 points, No. (%)	28 (12.7)	36 (16.4)	NA	.33
**PROMIS GMH**				
Baseline to 6 mo, No.	404	404	NA	NA
*T*-score				
Baseline	46.53 (8.97)	46.38 (9.00)	NA	.80
6 mo	47.84 (8.47)	46.62 (9.11)	NA	.04
Change	1.31 (6.66)^b^	0.24 (5.98)	+1.07 (0.62)	.02
Improve ≥5 points, No. (%)	109 (27.0)	81 (20.0)	NA	.02
Worsen ≥5 points, No. (%)	61 (15.1)	70 (17.3)	NA	.46
Baseline to 12 mo, No.	223	223	NA	NA
*T*-score				
Baseline	46.70 (9.21)	46.54 (12.12)	NA	.86
12 mo	47.22 (9.33)	46.73 (10.29)	NA	.58
Change	0.53 (7.03)	0.19 (7.15)	+0.34 (0.93)	.62
Improve ≥5 points, No. (%)	53 (23.8)	52 (23.3)	NA	.99
Worsen ≥5 points, No. (%)	46 (20.6)	54 (24.2)	NA	.42

Abbreviations: Center for Functional Medicine, Cleveland Clinic Center for Functional Medicine; Family Health Center, Cleveland Clinic Twinsburg Family Health Center; NA, not applicable; PROMIS GMH, Patient-Reported Outcome Measurement Information System Global Mental Health; PROMIS GPH, PROMIS Global Physical Health.

^a^ Primary outcome.

^b^ Statistically significant improvement within location group, *P* < .05; *P* value from paired *t* test and McNemar test.

Patients seen at the Center for Functional Medicine also had significant improvement in their mean (SD) PROMIS GMH scores at 6 months (from 46.53 [8.97] at baseline to 47.84 [8.47] at 6 months in 404 patients; *P* < .001), and the mean (SD) change was also significantly greater than that seen in the Family Health Center (Center for Functional Medicine: 1.31 [6.66] vs Family Health Center: 0.24 [5.98] *T*-score points in 404 patients; *P* = .02) (Table 3). In addition, more Center for Functional Medicine patients improved their PROMIS GMH scores by 5 or more points than the Family Health Center (Center for Functional Medicine: 109 [27.0%] vs Family Health Center: 81 [20.0%]; *P* = .02; number needed to treat, 14). Patients seen at the Center for Functional Medicine exhibited smaller, nonsignificant improvements in their PROMIS GMH scores at 12 months (from 46.70 [9.21] at baseline to 47.22 [9.33] at 12 months in 223 patients, *P* = .55), and the mean change was not significant compared with patients seen at the Family Health Center (Center for Functional Medicine: 0.53 [7.03] vs Family Health Center: 0.19 [7.15] *T*-score points in 223 patients; *P* = .62).

**Figure 2.**
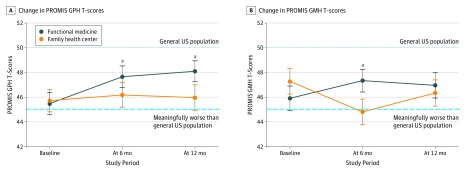
Continuous Change in Patient-Reported Outcome Measurement Information System (PROMIS) Global Physical Health (GPH) and Global Mental Health (GMH) *T*-Scores at 6 and 12 Months A, Continuous change in PROMIS GPH *T*-scores at baseline, 6 months, and 12 months in propensity score–matched patients (n = 91) in the functional medicine and family health care centers with scores at each time point. B, Continuous change in PROMIS GMH *T*-scores at baseline, 6 months, and 12 months in propensity score–matched patients (n = 97) in the functional medicine and family health care centers with scores at each time point. Change of 5 or more points was considered clinically meaningful on PROMIS Global Health *T*-score scales. Vertical lines represent SEs. ^a^Significant between-group differences, *P* < .05.

Figure 2 shows the results of the sensitivity analysis performed on patients with follow-up PROMIS GPH and GMH evaluation at both 6 and 12 months. Mean (SD) baseline PROMIS GPH scores were similar for the Center for Functional Medicine (45.49 [8.51]) and the Family Health Center (45.73 [8.62]) within 91 PS-matched pairs and were below the general US population mean (SD) score of 50.0 (10.0) (Figure 2A). At 6 months and 12 months, patients seen at the Center for Functional Medicine had significantly better mean (SD) PROMIS GPH scores at 6 (47.65 [8.32]) and 12 (48.10 [8.17]) months compared with those seen at the Family Health Center at 6 (46.19 [9.72]) and 12 (46.97 [9.82]) months (*P* = .049 at 6 months; *P* = .04 at 12 months). Patients in the functional medicine center with data at both 6 and 12 months demonstrated improvements in PROMIS GPH (mean [SD], 2.61 [6.53]) that were significantly larger compared with patients seen at a family health center (mean [SD], 0.25 [6.54]) (*P* = .02 in 91 PS-matched pairs). Mean (SD) baseline PROMIS GMH scores were also similar for both centers within 97 PS-matched pairs (Center for Functional Medicine: 45.92 [9.74]; Family Health Center: 47.29 [10.26]) and were also below the general US population mean score of 50 (Figure 2B). Patients seen at the Center for Functional Medicine only had significantly better PROMIS GMH scores at 6 months compared with those seen at the Family Health Center (Center for Functional Medicine: 47.35 [9.02] vs Family Health Center: 44.82 [10.18]; *P* = .049). Categorical improvements of PROMIS GPH scores from baseline to 12 months in patients with 6-month data are displayed in the eFigure in the Supplement.

The mean (SD) 6-month GPH score for patients with scores measured at 6 and 12 months (46.93 [9.05]; n = 182) was not statistically different from the mean (SD) 6-month GPH score for patients with scores at 6 but not 12 months (47.34 [8.31]; n = 664) (*P* = .58). Likewise, the mean (SD) 6-month GMH score for patients with scores at 6 and 12 months (46.55 [9.75]; n = 194) was not statistically different from the mean (SD) 6-month GMH score for patients with scores at 6 but not 12 months (47.40 [8.66]; n = 669) (*P* = .27).

## Discussion

To date, the evidence to support the functional medicine model of care has been anecdotal, primarily published as case reports.^23,24^ Peer-reviewed evidence for functional medicine is based on specific interventions used by the model, including nutrition,^25^ lifestyle,^26^ or medications and dietary supplements (monotherapy or polytherapy).^27^ To our knowledge, this study is also the first systematic attempt to collect data from patients using validated measures to understand the association of HRQoL with the functional medicine model of care.

In this study, the functional medicine model of care was significantly associated with improved longitudinal PROMIS GPH scores in patients at 6 months, and these improvements remained significant for up to 12 months. Patients seen at the Center for Functional Medicine were more likely to experience a clinically meaningful change (change of ≥5 points) in their PROMIS GPH scores at 6 months, which were less likely to decrease over time. Comparing PROMIS GPH scores with those from the Family Health Center, patients seen at the Center for Functional Medicine experienced a significant longitudinal benefit for up to 12 months. However, a more robust sample size and consistent longitudinal tracking of patients are warranted to confirm this finding. The functional medicine model of care also significantly improved short-term PROMIS GMH scores in patients and demonstrated a larger association than care received in a primary care setting; however, long-term improvements were not statistically significant.

Several factors may have contributed to improvements in HRQoL associated with the functional medicine model of care. First, improvements in HRQoL associated with the functional medicine model of care may be due to the model itself. Functional medicine addresses chronic disease by delivering precision medicine. The ability to deliver precision medicine relies on one’s capability to not only collect data, but also organize it in a way that extracts an understanding of a patient’s biological processes and then maps these processes to human disease.^28,29^ The delivery of precision medicine also requires the ability to focus treatment around specific factors associated with a patient’s symptoms. The formal definition of functional medicine was first introduced in 1991 and tracks with the more recent precision medicine initiative.^11,28,29^ The use of the word *function* within the name is “aligned with the evolving understanding that disease is an endpoint and function is a process.”^11^^(p25)^ The functional medicine model uses a systems-based approach to care that looks upstream of a patient’s symptoms and considers the complex web of interactions within a patient’s history, physiologic status, genetics, lifestyle, and environment, and contributes to their physical and mental functional status.^11^ The organization of this information within an operating system affords trained caregivers the opportunity to develop patient-specific management strategies to improve function through nutritional, behavioral, and lifestyle interventions. Studies have suggested an association between biological pathways, genes, and molecular markers and quality-of-life domains (eg, physical function, fatigue, pain, emotional function, social function, and overall quality of life).^30^

Although not inherent to all functional medicine practices, the Center for Functional Medicine requires that all new patients see a registered dietitian and health coach, in addition to a clinician, as part of their initial visit. Patients also have the option to meet with a behavioral health therapist as part of any visit. Dietitians and health coaches are integral because they address the nutritional, psychological, and social aspects of patients’ illnesses and promote long-term self-management, which are components needed for the treatment of various chronic conditions.^31^ This clinical operational structure is different from that delivered in conventional medicine where health coaches are not available and scheduling a visit with a registered dietitian may not be recommended and/or available. In addition, the findings reported herein may not be representative of other functional medicine private practices, because multidisciplinary teams are not ubiquitous.

Second, patients seen in the Center for Functional Medicine may be different from those seeking primary care in a family health center. Our attempt to circumvent this bias was to PS match patients from each center based on certain variables; however, there may be unmeasured confounders associated with the reported outcomes. For example, patients who request to be seen at the Center for Functional Medicine may be more motivated to make a nutrition-, lifestyle-, or behavior-related change in their life. Success with such change is associated with patient activation measures relating to engagement and self-management opportunities.^32,33,34^ Higher patient activation is also associated with individuals who perceive that they have an unmet need as it relates to their medical care.^33^ Patients seeking functional medicine may have exhausted all available opportunities in conventional medicine to manage or mitigate their chronic disease and perceive functional medicine as their only recourse. Therefore, patients seen in the functional medicine setting may be more engaged and adherent to treatment recommendations. Evidence also suggests that greater patient activation is associated with higher income and more education.^33^ However, the median income level for patients seeking functional medicine was significantly lower ($13 588 less) than for those seeking care in a family health center before PS matching.

In addition, there may be factors contributing to positive healing in patients receiving functional medicine care unrelated to the treatment received, including inherent patient bias toward the efficacy of the model of care, visits in a newer facility or at Cleveland Clinic main campus, or the duration of the initial patient visit. At the initial visit, patients have 60 to 75 minutes of clinician time compared with a much shorter duration in conventional care. Taken together, all of these considerations may have been associated with possible bias of patient-reported outcomes involved in this study.

Third, improvements in HRQoL associated with the functional medicine model of care may be owing to therapeutic partnerships that caregivers build with the patients that empower the patients to be stewards of their health. This process is a shift away from the traditional disease-focused approach to a patient-centered approach that uses the patient’s story to create lasting change. The cultivation of a therapeutic partnership between the patient and their caregivers begins at the initial visit, which is substantially longer than an initial visit in a primary care setting. In the functional medicine setting, trained caregivers connect with patients by developing a strong rapport, fostering open communication, and developing a healing language rooted in empathy.^35^ Therapeutic partnerships enable patients to become active participants in their care alongside their caregivers rather than bystanders, which may be associated with both satisfaction^36^ and outcomes most likely owing to altered self-management and adherence to therapies.^37^

Fourth, improvements in HRQoL associated with the functional medicine model of care may be owing to ascertainment bias whereby patients with follow-up at 6 and 12 months may be those improving owing to treatment adherence or belief in the model of care. Conversely, patients without follow-up may be less adherent or may not believe that functional medicine can help them. It is also possible that patients who did not complete long-term follow-up received benefit from the initial recommendations and felt better. There was no plan for gathering longitudinal data on patients without follow-up. Ultimately, patients without follow-up were excluded from the overall analysis. This bias may not be associated with patients seen at the Family Health Center, because they are receiving routine care or physicals vs study follow-up.

Future studies related to the functional medicine model of care would examine its delivery to determine how it may be associated with proximal (eg, patient and clinician satisfaction and treatment adherence) and distal (eg, symptom burden and total cost of care) outcomes.^37,38^ In addition, studies that examine outcomes related to the use of ancillary services provided by a dietitian and health coach (frequency and duration of visits and content discussion) are warranted.

### Limitations

There are several limitations to this study. First, PS matching of patients on several variables resulted in the loss of eligible patients. However, this step was necessary owing to differences in the patient populations. As a result, generalizations regarding PS-matched Functional Medicine and Family Health Center patients to all patients in those groups should be avoided. Second, despite various analyses, there were no adjustments for multiple comparisons. The results of our exploratory study are hypothesis generating and focused on magnitudes of differences rather than statistical significance. Third, we recognize that a nonresponse bias exists with respect to the longitudinal collection of PROMIS GPH and GMH scores at 12 months. Further analyses are warranted to evaluate longitudinal outcomes.

## Conclusions

The present study suggests that the functional medicine model of care may have beneficial and sustainable associations with improved HRQoL in patients as measured by PROMIS GPH and GMH scores. The use of PROMIS measures may provide timely information on a patient’s global health and could improve chronic disease management.

## Notes

Supplement. **eTable 1.** Comparisons of Patients Included in the Study Versus Excluded in Study **eTable 2.** Categorization of ICD-9/-10 Coding to Functional Medicine Diagnostic Categories **eFigure.** Categorical Change in PROMIS GPH T-Scores at 6 Months and 12 Months
