# Supplementary material for: Association of the Functional Medicine Model of Care With Patient-Reported Health-Related Quality-of-Life Outcomes
Source: JAMA Netw Open. 2019 Oct 25;2(10):e1914017. doi: 10.1001/jamanetworkopen.2019.14017 (PMC6822085; doi:10.1001/jamanetworkopen.2019.14017)
Supplement: Supplement. — eTable 1. Comparisons of Patients Included in the Study Versus Excluded in Study eTable 2. Categorization of ICD-9/-10 Coding to Functional Medicine Diagnostic Categories eFigure. Categorical Change in PROMIS GPH T-Scores at 6 Months and 12 Months [file jamanetwopen-2-e1914017-s001.pdf]

Supplementary Online Content

Beidelschies M, Alejandro-Rodriguez M, Ji X, Lapin B, Hanaway P, Rothberg MB. Association of the functional medicine model of care with patient-reported health-related quality-of-life outcomes. *JAMA Netw Open*. 2019;2(10):e1914017. doi:10.1001/jamanetworkopen.2019.14017

eTable 1. Comparisons of Patients Included in the Study Versus Excluded in Study

eTable 2. Categorization of ICD-9/-10 Coding to Functional Medicine Diagnostic Categories

eFigure. Categorical Change in PROMIS GPH T-Scores at 6 Months and 12 Months

This supplementary material has been provided by the authors to give readers additional information about their work.

© 2019 Beidelschies M et al. *JAMA Network Open.*

eTable 1. Comparisons of Patients Included in the Study Versus Excluded in Study

Characteristic	Included in Study Cohort N (%)	Excluded N (%)	P-Value
Total # of Patients	7252	4312	
Age, mean (SD), y	54.1 (16.0)	51.2 (15.9)	<0.001
Women	4780 (65.9)	2858 (66.3)	0.70
White race	6283 (87.0)	3630 (85.4)	0.01
Married	4753 (66.0)	2815 (65.8)	0.78
Household Income, median (IQR), $	66314.0 (53944.0, 79293.0)	67332.0 (53944.0, 81060.5)	0.03
Family Health Center	5657 (78.0)	3206 (74.4)	<0.001
Diabetes	2215 (30.5)	970 (22.5)	<0.001
Depression	1773 (24.4)	897 (20.8)	<0.001
Hypertension	3187 (43.9)	1416 (32.8)	<0.001
Baseline score, mean (SD)			
PROMIS GPH	47.86 (8.48)	48.11 (8.75)	0.13
PROMIS GMH	49.08 (9.31)	48.77 (9.51)	0.08
Total Visits, mean (SD), No.	4.39 (2.52)	2.60 (1.01)	<0.001
Functional Medicine Diagnostic Category*			
Infection	528 (7.3)	310 (7.2)	0.88
Autoimmune	725 (10.0)	438 (10.2)	0.81
Allergen	187 (2.6)	132 (3.1)	0.14
Cancer	358 (4.9)	180 (4.2)	0.07
Hormones	2045 (28.2)	1142 (26.5)	0.05
Energy Mitochondria	852 (11.7)	647 (15.0)	<0.001
Nutrition	96 (1.3)	30 (0.7)	0.002
Mood	173 (2.4)	100 (2.3)	0.87
Neurology	742 (10.2)	495 (11.5)	0.04
HEENT	187 (2.6)	119 (2.8)	0.60
CVD	1348 (18.6)	600 (13.9)	<0.001
Gut	1015 (14.0)	625 (14.5)	0.47
Skin	485 (6.7)	307 (7.1)	0.40
Structure	994 (13.7)	598 (13.9)	0.83
Genitourinary	709 (9.8)	406 (9.4)	0.55
Trauma	76 (1.0)	42 (1.0)	0.77

Abbreviations: IQR, interquartile range; Family Health Center, Cleveland Clinic Twinsburg Family Health Center; PROMIS, Patient-Reported Outcome Measurement Information System; GPH, Global Physical Health; GMH, Global Mental Health; HEENT, head, eyes, ears, nose, and throat; CVD, cardiovascular disease. *See eTable 2 in the Supplement.

© 2019 Beidelschies M et al. *JAMA Network Open.*

eTable 2. Categorization of ICD-9/-10 Coding to Functional Medicine Diagnostic Categories

Functional Medicine Diagnostic Category	Categorization of ICD-9/-10 Codes*
Infection	Certain infectious and parasitic diseases
Autoimmune	Diseases of the immune system caused by antibodies or autoantibodies
Allergen	Diseases or adverse effects due to poisoning or external agents
Cancer	Neoplasms, diseases of the blood and blood-forming organs and certain disorders involving the immune mechanism
Hormones	Endocrine diseases
Energy Mitochondria	Mitochondrial diseases
Nutrition	Nutritional and metabolic diseases
Mood	Mental, behavioral and neurodevelopmental disorders
Neurology	Diseases of the nervous system
HEENT	Diseases of the eye, adnexa, ear and mastoid process
CVD	Diseases of the circulatory and respiratory systems
Gut	Diseases of the digestive system
Skin	Diseases of the skin and subcutaneous tissue
Structure	Diseases of the musculoskeletal system and connective tissue
Genitourinary	Diseases of the genitourinary system
Trauma	Injury, poisoning and certain other consequences of external causes

Abbreviations: HEENT, head, eyes, ears, nose, and throat; CVD, cardiovascular disease.

*A detailed list of itemized ICD-9/-10 codes for each diagnostic category is available upon request.

© 2019 Beidelschies M et al. *JAMA Network Open.*

eFigure. Categorical Change in PROMIS GPH T-Scores at (a) 6 months and (b) 12 months

a. b.

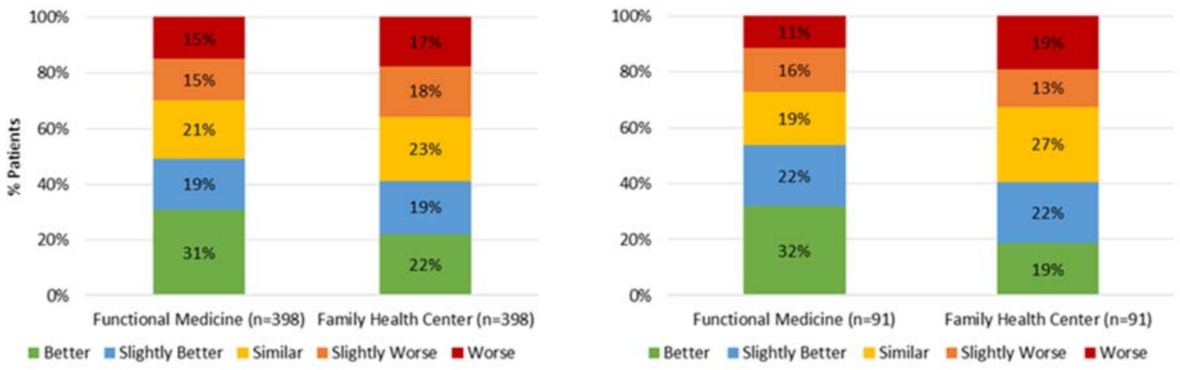

a. Change in PROMIS GPH T-scores at 6 months, b. Change in PROMIS GPH T-scores at 12 months, in PS-matched patients with scores at 6 months

"Better" defined as patients with improvement of 5+ points on the T-score scale; "Slightly Better" defined as improvement of between 2.5-5 points; "Similar" as change less than 2.5 points; "Slightly worse" as worsening between 2.5-5 points; "Worse" as worsening ≥5 points.

Change of 5+ points considered clinically meaningful on PROMIS Global Health T-score scales.

GPH = Global Physical Health

© 2019 Beidelschies M et al. *JAMA Network Open.*
